# Effects of Cocoa Genotypes on Coat Color, Platelets and Coagulation Parameters in French Bulldogs

**DOI:** 10.3390/genes12071092

**Published:** 2021-07-19

**Authors:** Anna Laukner, Laura Truchet, Georgi Manukjan, Harald Schulze, Ines Langbein-Detsch, Elisabeth Mueller, Tosso Leeb, Alexandra Kehl

**Affiliations:** 1 Laboklin GmbH&Co.KG, Steubenstraße 4, 97688 Bad Kissingen, Germany; dr.laukner@gmx.de (A.L.); truchet@laboklin.com (L.T.); langbein@laboklin.com (I.L.-D.); mueller@laboklin.com (E.M.); 2 Institute of Experimental Biomedicine, University Hospital Wuerzburg, 97074 Würzburg, Germany; georgimanukjan@hotmail.com (G.M.); harald.schulze@uni-wuerzburg.de (H.S.); 3 Institute of Genetics, Vetsuisse Faculty, University of Bern, 3001 Bern, Switzerland; tosso.leeb@vetsuisse.unibe.ch

**Keywords:** *Canis lupus familiaris*, dog, thrombocyte, pigmentation, hematology, platelet

## Abstract

A nonsense variant in *HPS3*, c.2420G>A or p.Trp807*, was recently discovered as the cause for a brown coat color termed cocoa in French Bulldogs. Here, we studied the genotype–phenotype correlation regarding coat color in *HPS3* mutant dogs that carried various combinations of mutant alleles at other coat color genes. Different combinations of *HPS3, MLPH* and *TYRP1* genotypes resulted in subtly different shades of brown coat colors. As *HPS3* variants in humans cause the Hermansky–Pudlak syndrome type 3, which in addition to oculocutaneous albinism is characterized by a storage pool deficiency leading to bleeding tendency, we also investigated the phenotypic consequences of the *HPS3* variant in French Bulldogs on hematological parameters. *HPS3* mutant dogs had a significantly lowered platelet dense granules abundance. However, no increased bleeding tendencies in daily routine were reported by dog owners. We therefore conclude that in dogs, the phenotypic effect of the *HPS3* variant is largely restricted to pigmentation. While an effect on platelet morphology is evident, we did not obtain any indications for major health problems associated with the cocoa coat color in French Bulldogs. Further studies will be necessary to definitely rule out very subtle effects on visual acuity or a clinically relevant bleeding disorder.

## 1. Introduction

Coat color is an important attribute of dogs and human preferences for specific coat colors are subject to trends. In recent years, brown French Bulldogs have become more and more popular. Brown pigmentation in dogs can be caused by at least six different variants in the *TYRP1* gene, which corresponds to the B locus from classical genetics [1,2,3,4]. Three of these mutant *TYRP1* alleles are widespread and segregate in many breeds, whereas the other three mutant *TYRP1* alleles appear to be much younger and restricted to specific breeds. In a recent study, the *HPS3*:c.2420G>A nonsense variant was found as an additional underlying genetic cause for the brown coat color in French Bulldogs. To distinguish it from the *TYRP1*-related phenotype, the resulting color in *HPS3* mutant dogs was named cocoa [5]. *TYRP1*-related brown and *HPS3*-related cocoa are recessive traits. The wildtype alleles at the *TYRP1* and *HPS3* genes are often abbreviated as *B* and *Co*, while the recessive mutant alleles are represented as *b* and *co*. The cocoa coat color is slightly darker than the *TYRP1*-related brown coat color in French Bulldogs [1,5].

Variants in the *HPS3* gene in humans were reported to cause the Hermansky–Pudlak syndrome type 3 (HPS3). This rare autosomal recessive disorder is characterized by oculocutaneous albinism and a bleeding diathesis due to the absence of platelet dense granules [6]. Patients also show mild nystagmus and mildly reduced visual acuity [7]. In mice, a similar phenotype with a brown coat color and prolonged bleeding time is known as cocoa mouse mutant and caused by a genetic variant in the murine *Hps3* gene [8,9].

The aim of this study was to characterize the phenotypic effect of the canine *co* allele on coat color in combination with different genotypes at other coat color genes: *tyrosinase related protein 1*(*TYRP1), melanophilin (MLPH), melanocortin 1 receptor (MC1R), agouti signaling protein (ASIP), major facilitator superfamily domain containing 12 (MFSD12), premelanosome protein (PMEL*). We also investigated whether the cocoa phenotype in French Bulldogs has additional phenotypic consequences on thrombocyte function and coagulation parameters.

## 2. Materials and Methods

### 2.1. Animals and Test Material

The study animals included 34 privately owned French Bulldogs with different coat colors. Blood samples consisting of whole blood anticoagulated with EDTA and sodium citrate were drawn for diagnostic health checks after obtaining owners’ consent. Leftover blood samples were used for the genetic screening and a kinetically resolved mepacrine uptake and release test was used as a surrogate for platelet dense granule abundance and release properties. Testing was performed within 24 h after taking samples. Photographs of dogs 1–13 were taken by AL with a digital camera within one afternoon and under the same technical conditions. Photographs of dogs 23–29 were taken by the owners. Photographs were available for 20 dogs (5 *co*/*co*, 9 *Co*/*co* and 6 *Co*/*Co*).

### 2.2. Clinical Parameters

A complete blood count was performed with EDTA-anticoagulated whole blood using the Sysmex XT2000 (Sysmex, Kobe, Japan). Samples were thoroughly inverted and then analyzed as recommended by the manufacturer.

Thromboelastography (TEG) was performed with recalcified citrate whole blood using the TEG 5000 Thromboelastograph Hemostasis Analyzer (Haemonetics GmbH, Boston, MA, USA). The cup was placed according to the manufacturer’s instructions and 20 µL of 0.2 M CaCl2 was pipetted in the cup. One milliliter of the citrate whole blood was transferred to a kaolin tube (Haemonetics GmbH) and mixed by gentle inversion 5 times. Immediately afterwards, 340 µL of the kaolin citrate whole blood mixture was pipetted to the CaCl2 solution in the cup and the thromboelastograph was started.

The coagulation parameters, prothrombin time (PT), activated partial thrombin time (aPTT) and thrombin time, were analyzed using the STA Compact Max3 (Asnières-sur-Seine, France) according to the manufacturer’s instructions.

Dog breeders/owners were contacted via phone and asked for conspicuities regarding bleeding tendencies and vision.

### 2.3. Mepacrin Assay

The mepacrine uptake and release assay in whole blood was assessed by measuring a 60 s baseline fluorescence of resting platelets on a FACSCelesta flow cytometer (Becton Dickinson, Heidelberg, Germany). Platelets were then loaded with mepacrine dihydrochloride dye (Sigma-Aldrich, Darmstadt, Germany) to a final concentration of 25 µM for 30 min at 37 °C and the mepacrine-based fluorescence after sub-gating to CD61-positive platelets (antibody clone Y2/51; Bio-Rad Laboratories, Feldkirchen, Germany) was recorded for further 60 s. Stained platelets were activated using 0.1 U/mL thrombin from human plasma (Roche, Freiburg, Germany) and directly analyzed for additional five minutes. The difference between unloaded and loaded as well as loaded and released platelets was calculated as deltaMFI (mean fluorescence intensity) [10].

### 2.4. Genotyping

Genomic DNA was isolated from EDTA blood samples using MagNAPure (Roche, Basel, Suisse) with the MagNA Pure DNA and viral NA kit according to the manufacturer’s instructions. Genotyping for Cocoa, A, B, D, E and I loci was performed by using TaqMan SNP Assay (LifeTechnologies, Carlsbad, CA, USA) specific to the related variant and LC480 (Roche, Basel, Suisse). Genotyping for the SINE insertions black and tan (at) phenotypes was performed by fragment length analysis on an ABI Genetic Analyser 3130 (LifeTechnologies, Carlsbad, CA, USA) after amplification of the region of interest with FAM-marked primers. Genotyping for Merle was carried out by Laboklin sro, Bratislava. Details of the tested variants are listed in Table S1.

## 3. Results

### 3.1. Coat Colors

**Figure 1:**
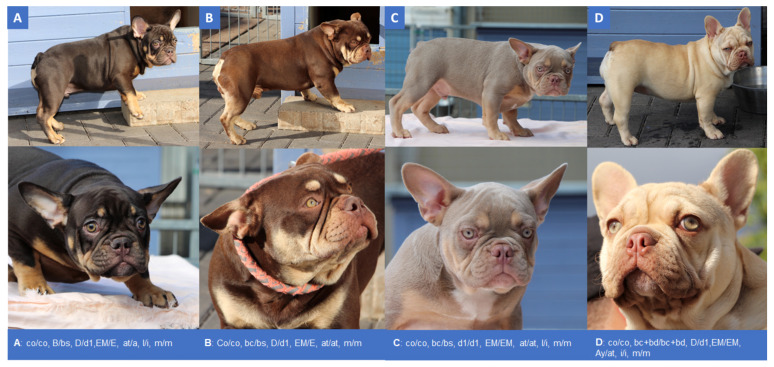

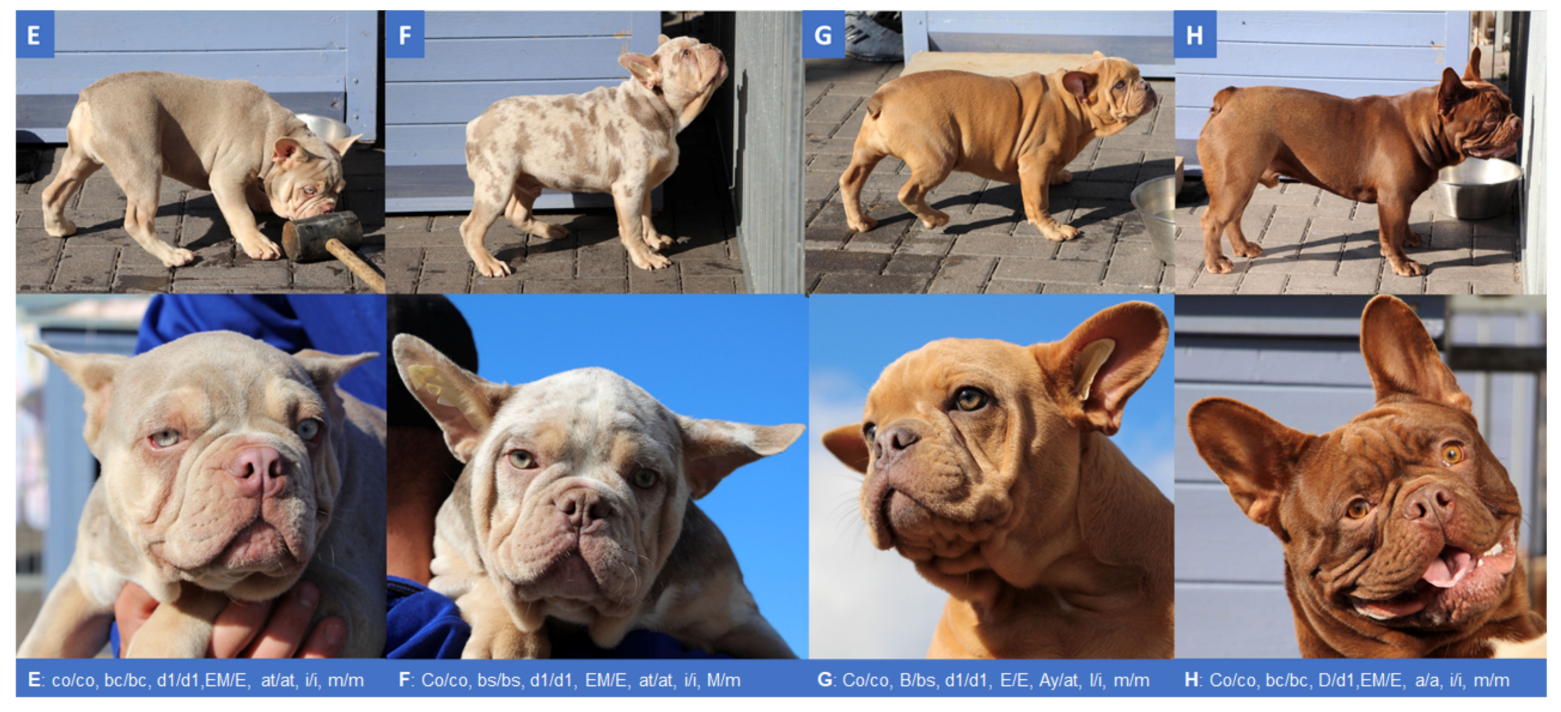
Different coat color and pattern phenotypes of French Bulldogs, depending on genotypes at *HPS3* in combination with different genotypes at *TYRP1, MLPH, MC1R, ASIP, MFSD12* and *PMEL* genes. Genotypes at the underlying loci are indicated (see Table S1). (**A**) Cocoa and tan dog with dark brown eumelanin, yellow pheomelanin markings, dark brown nose and light brown eyes. The muzzle is dark brown, due to dominant *EM* allele at *MC1R*. Note the intense yellow markings; pheomelanin has not been diluted by *co*/*co*. (**B**) *TYRP1*-related brown and tan dog with brown eumelanin, light yellow pheomelanin markings, brown nose and yellow eyes. (**C**) Dilute cocoa plus brown and tan dog with greyish-brown eumelanin, yellow pheomelanin markings, greyish-brown nose and light green eyes. (**D**) Pale yellow dog due to the dominant yellow *Ay* allele at the *ASIP* gene. Cocoa plus brown eumelanin can be seen around the muzzle due to the presence of *EM* at *MC1R*. The dog has a greyish-brown nose and yellow eyes. (**E**) Dilute cocoa plus brown and tan dog with greyish-brown eumelanin, pale yellow pheomelanin markings, greyish-brown nose and light green eyes. Note the slightly paler markings than in C, due to the genotype *i/i* at the *MFSD12* gene. (**F**) Dilute brown and tan dog with pale yellow markings, greyish-brown nose and light green eyes. Greyish-brown muzzle due to *EM* at *MC1R*. Eumelanin is further diluted in random areas, creating a pattern of greyish-brown patches in different shades on body, upper parts of legs, neck and head, due to the presence of the dominant Merle allele (*M*) at *PMEL*. (**G**) Intense yellow dog with greyish nose and light brown eyes. Compare the intense pheomelanin color to the pale yellow of D, due the genotype *i*/*i* at *MFSD12*. (**H**) *TYRP1*-related brown dog with brown nose and brown eyes. Phenotypical differentiation between *co/co B/-* (cocoa) and *Co/- b/b* (brown) is most reliable in dogs with a large proportion of eumelanin (e.g., black and tan or solid black base color, see A, B and H) and no other eumelanin diluting and/or attenuating genotypes (e.g., *d*/*d* at *MLPH*). Coat, nose and eye color phenotypes are influenced by different gene loci. The genotype *b*/*b d*/*d* affected the phenotype of *co*/*co* dogs by further diluting eumelanin from dark brown to greyish brown (**C**,**E**,**F**). Note: It is not possible to reliably differentiate *co/co* from *Co/-* phenotypically, if further eumelanin reducing (e.g., *Ay* at *ASIP*) (see (**D**,**G**)) and/or eumelanin attenuating (especially *TYRP1* and/or *MLPH*) (see (**C**,**E**–**H**)) alleles are present.

We obtained photographs from 20 French Bulldogs with different coat color genotypes, including five *co*/*co* dogs (Figure 1, Table S2). We confirmed the earlier observation that the eumelanin coat color in adult cocoa dogs (*co*/*co*) is of a darker brown shade than the eumelanin coat color in *TYRP1* deficient dogs (*b*/*b*) (Figure 1A,B) [5]. However, the data also illustrate an enormous complexity of additive and/or epistatic gene–gene interactions of *HPS3* and *TYRP1* with other coat color genes. It is often not possible to deduce the underlying genotype from the phenotype. At the same time, genotyping only the *HPS3* gene alone does not allow one to make reliable predictions of the coat color of a French Bulldog. The comprehensive characterization of the coat color of a French Bulldog often requires the genotyping of several coat color loci.

### 3.2. Hematological Investigations

Complete blood counts were mostly without any particular findings (Table S3). Mild leukocytosis was seen almost exclusively in young dogs at the age of six months (*n* = 10). In this group, 60% of the dogs showed mild absolute lymphocytosis and about 40% showed very mild to mild absolute neutrophilia, monocytosis and partially mild eosinophilia. A mild thrombocytosis was seen in five dogs, also mainly in the younger population (six to eleven months). Very few changes were seen in the examination of the PT, aPTT and thrombin time. All three parameters were mildly prolonged in one dog. Four dogs showed a very mildly prolonged aPTT. Two of the latter additionally showed very mildly to mildly prolonged PT and one of those showed a normal aPTT when examined a second time. The thromboelastography discovered four cases of hypocoagulopathy in the dogs older than 16 months and seven cases of hypercoagulopathy in the puppies of up to six months. Of the latter, three were very mild hypercoagulopathies. None of these findings showed a significant correlation with the genotypes at the *HPS3* gene.

**Figure 2:**
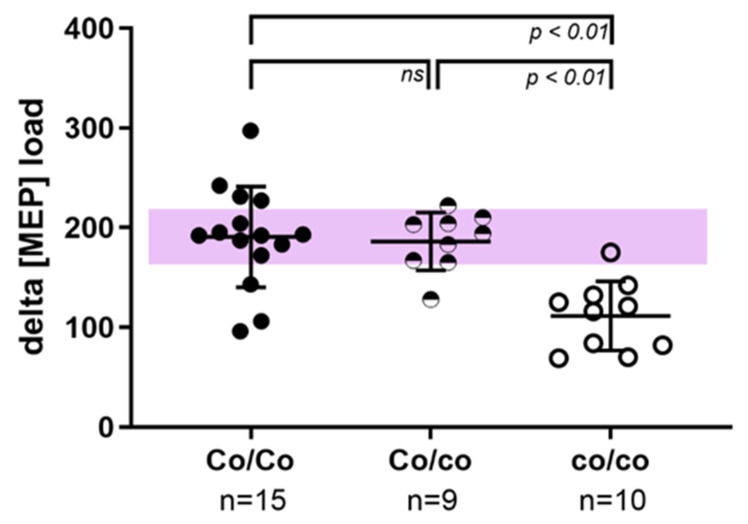
Platelet storage pool deficiency in cocoa dogs. Platelets of cocoa dogs (*co*/*co*; *n* = 10, white ball) present with a clearly reduced mepacrine loading capacity indicative of a lack of platelet dense granules. Heterozygous carriers (*Co*/*co*; *n* = 9, half ball) show mepacrine loading values comparable to wildtype animals (*Co*/*Co*; *n* = 15, black ball). Purple box indicates 95% confidence interval of the wildtype group. Non-parametric one-way ANOVA according to Kruskal–Wallis was used to calculate statistical significance (ns = no significance).

To study a possible influence of the *HPS3* variant on platelet dense granule abundance, we performed a kinetic mepacrine assay. Mepacrine is an antibiotic that spontaneously incorporates itself into platelet dense granules and can be released upon stimulation of platelets in response to multiple/distinct agonists [11]. Due to its intrinsically fluorescent capacity, mepacrine fluorescence can easily be detected and recorded by flow cytometry, for instance, and hence is a reliable marker to detect storage pool deficiencies [10]. In *co/co* dogs (*n* = 10), mepacrine load was overall significantly decreased compared to dogs with the genotypes *Co*/*Co* (*n* = 15) or *Co*/*co* (*n* = 9) (Figure 2). This indicates a recessive dense granule defect in those animals. No coagulation problems were reported by owners of the dogs included in this study.

## 4. Discussion

In this study, we investigated the phenotypic consequences of the *HPS3^co^* allele causing the cocoa coat color phenotype in French Bulldogs. In humans, the most obvious sign of Hermansky–Pudlak syndrome type 3 (HPS3) is the hypopigmentation of the skin and hair, leading to a mild form of oculocutaneous albinism. Furthermore, HPS3 in humans is characterized by ocular findings such as nystagmus, reduced visual acuity and a mild bleeding tendency caused by the absence of platelet dense granules [6,7].

First, we characterized the pigmentation phenotype. A great variety of distinct coat color phenotypes was observed, due to different genotypes at seven different color loci. By determining the variants of just one gene, no coat color phenotype can be predicted. Cocoa in French Bulldogs can be identified phenotypically, but only if no further eumelanin attenuating variants at *TYRP1* and/or *MLPH* and *PMEL* are present and if the base color is solid eumelanin or eumelanin and tan. Phenotypical differentiation between cocoa and brown eumelanin is possible in some cases due to the slightly darker color intensity of cocoa but might be difficult for an inexperienced viewer. In the context of our study, no final assessment could be made regarding epistasis between *HPS3* and *TYRP1*, because there was no dog with the genotype *co*/*co b*/*b* with a solid eumelanin or tanpoint coat color among the recruited dogs. The brown mask of dog 8 (Figure 1D) appeared lighter than the brown mask of dog 2 (Figure 1A), which may indicate epistasis of *b*/*b* over *co*/*co*. Due to the large number of different coat color alleles that segregate in French Bulldogs, it is often not possible to unambiguously determine the underlying genotype based on the phenotype alone. In many cases, genetic testing of several coat color loci is necessary to accurately determine the genotype of a dog. All coat colors produced by variants on *HPS3*, *TYRP1*, *MLPH*, *PMEL* and/or the genotypes *at*/*at*, *at*/*a* and *a*/*a* on *ASIP* are not recognized by the official FCI, AKC and KC breed standards of the French Bulldog.

Second, laboratory tests were performed and breeders/owners were asked for conspicuities regarding bleeding tendencies and vision. Clinical evidence of bleeding diathesis or of visual impairments was not reported. As the signs concerning bleeding disorders of HPS3 in humans are mild, it cannot be excluded that subtle clinical signs in dogs were overlooked. Therefore, we determined clinical parameters for increased bleeding tendency including PT, aPTT and thrombin time, TEG and complete blood count. No gross changes indicating bleeding diathesis were seen in cocoa dogs in all clinical parameters investigated. For these clinical parameters, values in the reference range were expected as these parameters are in reference ranges in humans too [12,13].

For additional data on bleeding tendencies in the examined dogs, a buccal mucosal bleeding time should be performed since the bleeding time is also significantly prolonged in humans, even if the other coagulation tests mentioned above are in the reference range [12,14]. Regarding the hypercoagulopathies in the results, it is worth mentioning that these only occurred in young dogs. This has already been described in a few cases in the literature. Furthermore, brachycephaly plays a role in hypercoagulopathies [15,16].

For diagnosis of HPS3 in humans, investigation of platelet dense granules via electron microscopy is considered to be the gold standard [17]. The mepacrine assay by flow cytometry was introduced as a more rapid and cost-effective alternative to electron microscopy [10,18]. A significant reduction in mepacrine load was observed in homozygous co/co dogs, probably caused by the lack of dense granules. One would expect that dogs with reduced mepacrine binding are prone to prolonged bleeding, as seen in humans. However, neither breeders, owners nor veterinarians reported any bleeding incident in a co/co dog in this study. Unlike in humans, the lack of platelet dense granules does apparently not lead to bleeding diathesis. Of note, the platelet dysfunction described for human patients with HPS3 also causes only a mild bleeding tendency that can nonetheless result in severe bleeding problems in response to trauma [19] or certain types of surgical procedures including tooth extraction, adenoidectomy or tonsillectomy. Given the sparse data, it cannot yet be excluded with certainty that very mild bleeding tendencies might be overlooked by owners. Additionally, no dog of the examined co/co cohort underwent any surgical procedure so far. For confirmation that the storage deficiency is clinically not relevant in dogs, the buccal mucosal bleeding time should be performed in a future study.

## 5. Conclusions

In conclusion, we provided comprehensive genotype–phenotype correlations in French Bulldogs with various combinations of genotypes at the *HPS3*, *TYRP1*, *MLPH*, *MC1R, ASIP, MFSD12* and *PMEL* genes. We also showed a significant impact of the *HPS3* variant on the function of the dense granules in platelets via mepacrine testing in accordance with findings in humans. In contrast to humans, the identified *HPS3* variant presumably does not lead to significantly increased bleeding tendencies in dogs though. As long as bleeding diathesis cannot be definitely excluded in *co/co* dogs by further studies, we recommend evaluating the buccal mucosal bleeding time before scheduled surgical interventions.

## Data Availability

Not applicable.

## Supplementary Materials

The following are available online at https://www.mdpi.com/article/10.3390/genes12071092/s1, Table S1. Details of the coat color variants tested in this study, Table S2. Coat color genotypes and available photographs of the dogs investigated, Table S3. Clinical parameters of the study population of 34 clinically healthy French Bulldogs ranging from two months to nine years.
